# PPGFeat: a novel MATLAB toolbox for extracting PPG fiducial points

**DOI:** 10.3389/fbioe.2023.1199604

**Published:** 2023-06-09

**Authors:** Saad Abdullah, Abdelakram Hafid, Mia Folke, Maria Lindén, Annica Kristoffersson

**Affiliations:** School of Innovation, Design and Engineering, Mälardalen University, Västerås, Sweden

**Keywords:** photoplethysmography, PPG features, fiducial points, MATLAB, toolbox, signal processing, acceleration photoplethysmography, velocity photoplethysmography

## Abstract

Photoplethysmography is a non-invasive technique used for measuring several vital signs and for the identification of individuals with an increased disease risk. Its principle of work is based on detecting changes in blood volume in the microvasculature of the skin through the absorption of light. The extraction of relevant features from the photoplethysmography signal for estimating certain physiological parameters is a challenging task, where various feature extraction methods have been proposed in the literature. In this work, we present PPGFeat, a novel MATLAB toolbox supporting the analysis of raw photoplethysmography waveform data. PPGFeat allows for the application of various preprocessing techniques, such as filtering, smoothing, and removal of baseline drift; the calculation of photoplethysmography derivatives; and the implementation of algorithms for detecting and highlighting photoplethysmography fiducial points. PPGFeat includes a graphical user interface allowing users to perform various operations on photoplethysmography signals and to identify, and if required also adjust, the fiducial points. Evaluating the PPGFeat’s performance in identifying the fiducial points present in the publicly available PPG-BP dataset, resulted in an overall accuracy of 99% and 3038/3066 fiducial points were correctly identified. PPGFeat significantly reduces the risk of errors in identifying inaccurate fiducial points. Thereby, it is providing a valuable new resource for researchers for the analysis of photoplethysmography signals.

## 1 Introduction

Photoplethysmography (PPG) detects changes in blood volume in the microvasculature of the skin through the absorption of light. The non-invasive technique is used to measure vital signs (Allen, 2007) and has been described in studies as a tool for monitoring heart rate, blood pressure, and respiratory rate (Fukushima et al., 2012; Karlen et al., 2013; He et al., 2014; Islam et al., 2017), and to identify persons with risk of diseases (Mahri et al., 2017; Chakraborty et al., 2020; Charlton et al., 2022b; Lee et al., 2022). However, extracting relevant features from the PPG signal is a challenging task (Elgendi et al., 2018; Chakraborty et al., 2019; Ab Hamid and Nayan, 2020).

The PPG waveform has been widely studied for a long time. Various feature extraction methods have been proposed in the literature (Elgendi et al., 2018; Chakraborty et al., 2019; Ab Hamid and Nayan, 2020). PPG is included in several wearable devices due to its non-complex setup and usefulness in the collection of a number of different and valuable physiological parameters (Almarshad et al., 2022). Despite this fact, there exists no agreed upon or standardized preprocessing framework allowing the use of the PPG signal for wearable applications without limitations (Prieto-Avalos et al., 2022). From choice of site for PPG signal acquisition to the features that can be extracted from the PPG signal, researchers have used various combinations of sensor interfacing, data recording, segmentation, filtration, and smoothing techniques (Chan et al., 2019).

There are several approaches for extracting PPG features in order to estimate different physiological parameters and vital signs (Khalid et al., 2018; Chen et al., 2019; Priyadarshini et al., 2021). One approach is the use of time- and/or frequency-domain analysis (Takada et al., 1996; Baek et al., 2010; Gil et al., 2010). Jaafar et al. (Jaafar and Rozali, 2018; Jaafar and Chung Xian, 2021) used only the time-domain of the PPG signal to estimate heart rate and breathing rate parameters. In another study, frequency-domain methods such as the Taguchi method (Suzuki and Ryu, 2014) were used in addition to time-domain analysis, in order to identify features which are relevant when estimating systolic blood pressure (Suzuki and Ryu, 2014).

To perform time-domain analysis, the velocity photoplethysmography (VPG), the acceleration photoplethysmography (APG), and the jerk photoplethysmography (JPG) are used. VPG, APG, and JPG correspond to the first, second and third PPG derivative, respectively.

In addition to the aforementioned feature extraction methods, the application of machine learning techniques has shown accurate and promising results and therefore seems to be an effective approach in extracting relevant features from PPG signals (El-Hajj and Kyriacou, 2020). Advanced signal processing techniques have also been used to extract relevant features for the estimation of blood pressure and other vital signs. An example of such a method is the Empirical Mode Decomposition (EMD) method (Vadrevu and Manikandan, 2017), which has been used to decompose PPG signals into a set of intrinsic mode functions (IMFs). The use of Discrete Wavelet Transform (DWT) has also been proposed and acceptable results have been achieved when used in the analysis of PPG signals (Paradkar and Chowdhury, 2015; Ricardo Ferro et al., 2015; Argüello Prada and Serna Maldonado, 2018).

Elgendi et al. (Elgendi et al., 2018) have attempted to standardize the nomenclature of the PPG, VPG and APG fiducial points. However, there is currently a lack of a widely accepted standard for the automatic detection of PPG fiducial points and its derivatives. Table 1 provides a general description of the main fiducial points in the PPG, VPG and APG.

**TABLE 1:** Characteristic points of the PPG signal and its derivatives.

Waveform	Fiducial points	Description
PPG	O	Onset
	S	Systolic peak
	N	Dicrotic notch
	D	Diastolic peak
VPG	w	Global maxima in the systolic phase
	x	Local minima in the systolic phase
	y	Global minima in the systolic phase
	z	First local maxima in the diastolic phase
APG	a	Early systolic positive peak
	b	Early systolic negative peak
	c	Late systolic re-increasing wave
	d	Late systolic re-decreasing wave
	e	Early diastolic positive wave
	f	Diastolic negative wave

**FIGURE 1:**
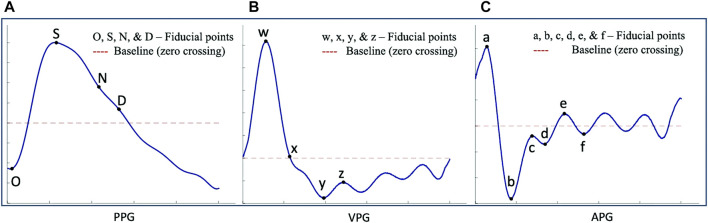
Illustration of fiducial points, where the blue lines in **(A)** represent the PPG waveform, **(B)** represent the VPG waveform, and **(C)** represent the APG waveform (Abdullah et al., 2023).

The PPG waveform (Figure 1A) consists of four main points of reference: the onset (O), the systolic peak (S), the dicrotic notch (N), and the diastolic peak (D). These points represent specific stages of the cardiac cycle, such as the beginning of the pulse in the systolic phase (onset) and the maximum peak during systolic ejection (systolic peak). The dicrotic notch (N) marks the transition from systole to diastole, while the diastolic peak (D) represents the minimum pressure in the arterial system. As individuals age, the dicrotic notch and diastolic peaks may become less pronounced in the PPG waveform (Mejía-Mejía et al., 2022), the APG waveform’s e and f points correspond to N and D.

The VPG waveform (Figure 1B) represents the velocity of the amplitude changes over time. It has two prominent peaks (w and y) in the systolic phase and one peak (z) in the diastolic phase. These peaks correspond to the points of maximum and minimum slope in the systolic and diastolic phases, respectively. Additionally, there is a local minimum (x) in the systolic phase which corresponds to the systolic peak of the PPG. The APG waveform (Figure 1C) provides additional information by including four prominent peaks in the systolic phase (a, b, c, and d) and two in the diastolic phase (e and f). These peaks correspond to the points of maximum acceleration in the waveform during the systolic and diastolic phases, respectively, and provide additional information about the dynamics of blood flow during these phases of the cardiac cycle (Charlton et al., 2018). Suboh et al. (Suboh et al., 2022) reported on the use of the third and fourth derivatives of the PPG waveform for detecting fiducial points in individuals with ischemic heart disease. However, Suboh et al. did not find the c and d points in all the waveforms they analyzed and thereby their features tables is incomplete.

In contrast, Abdullah et al. (Abdullah et al., 2023) have proposed an accurate method for classifying and finding the c and d points of the APG. This is significant progress because the statistical analysis of APG fiducial points can offer insights into arterial stiffness, aging, and essential hypertension (Takada et al., 1996; Brillante et al., 2008; Gil et al., 2010; Mok Ahn, 2017; Elgendi et al., 2019; Sarhaddi et al., 2022).

To facilitate research, open-source toolboxes for PPG have been developed and evaluated. Examples of these toolboxes are PPG-beats, PPGI, PPGTempStitch, and PhysioNet’s Cardiovascular Signal Toolbox and HRV Toolkit.

PPG-beats (Charlton et al., 2022a) is a MATLAB library which provides algorithms for detecting heartbeats from PPG-signals. It also provides a framework for assessing the performance of PPG heartbeat detectors in eight publicly available datasets containing both PPG and electrocardiogram (ECG) data. These are further described in Section 2.1.

PPGI (Pilz et al., 2019) is a MATLAB toolbox for PPG imaging which takes face videos as input for PPG and incorporates computer vision algorithms in order to estimate heart rate.

PPGTempStitch (Tang et al., 2021) is a MATLAB toolbox that takes annotated PPG waveforms as input and generates longer PPG waveforms simulating regular, irregular, fast rhythm, and noisy PPG waveforms. Two methods, which are based on the systolic peak and the onset respectively, are used for stitching the waveforms.

The PhysioNet Cardiovascular Signal Toolbox (Goldberger et al., 2000; Vest et al., 2018) is a toolbox for calculating heart rate variability, whereas the PhysioNet HRV Toolkit (Niskanen et al., 2004) allows for visualizing NN interval time series, automated removal of outliers and calculation of commonly used heart rate variability statistics.

However, we have not identified a toolbox aimed to identify fiducial points. In this article, we present PPGFeat, a robust, accurate and automated MATLAB based PPG feature extraction toolbox. PPGFeat has been developed and evaluated using a publicly available dataset that contains PPG waveforms collected from a diverse population including both healthy individuals and individuals with cardiovascular disease or other pathological disorders. The data was collected using a finger probe. The PPGFeat toolbox filters and examines the PPG waveform and its derivatives, provides the possibility to identify fiducial points from the PPG, VPG and APG, and generate a features table which can be further used for the statistical and AI based predictive analysis.

## 2 Materials and methods

### 2.1 Dataset selection

PPG-beats (Charlton et al., 2022a) includes the following eight publicly available datasets in their performance framework: CapnoBase IEEE TBME RR benchmark dataset, BIDMC, MIMIC PERform Training Dataset, MIMIC PERform Testing Dataset, MIMIC PERform AF Dataset, MIMIC PERform Ethnicity Dataset, WESAD, and PPG-DaLiA.

CapnoBase(CapnoBase IEEE TBME Respiratory Rate Benchmark - UBC Library Open Collections; Karlen et al., 2013) and BICMC(Goldberger et al., 2000; Pimentel et al., 2017) contain high-quality data collected during hospital monitoring. The patients in CapnoBase were 29 children and 13 adults receiving anesthesia whereas the 53 patients in BICMC were critically ill. The MIMIC PERform Training, Testing and AF datasets (Charlton et al., 2022a), which are subsets of Physionet’s MIMIC II dataset, also contain data from critically ill and hospitalized patients but the data is of lower quality due to being collected in routine clinical care. The MIMIC PERform training and testing datasets contain PPG, ECG, and respiration signals from 100 adults and 100 neonates whereas the MIMIC PERform AF dataset contains similar signals from 19 patients with atrial fibrillation and 16 patients with normal sinus rhythm. The MIMIC PERform Ethnicity Dataset (Charlton et al., 2022a), which is a subset of Physionet’s MIMIC II matched waveform dataset, contains data from 100 white and 100 black patients who were critically ill.

Three other datasets containing data from patients are The University of Queensland Vital Signs dataset (Saeed et al., 2011; Liu et al., 2012), which is another subset of PhysioNet’s MIMIC II dataset, and the PPG-BP dataset (Liang et al., 2018a). A wide range of data collected from 32 patients undergoing surgery with anaesthesia is included in the University of Queensland dataset (Liu et al., 2012), whereas the MIMIC II dataset (Saeed et al., 2011) contains ECG, respiratory rate, arterial blood pressure, and PPG signals from 90 patients admitted to an intensive care unit. Certain records in the MIMIC II dataset (Saeed et al., 2011) include systolic peak annotations. The PPG-BP dataset (Liang et al., 2018a) contains data from 219 patients of equal gender distribution who were admitted to the Guilin People’s Hospital in Guilin, China. The age of the patients was 21–86 years with a median age of 58 and they had several different diseases. PPG-BP integrates identified and comprehensive clinical data which allows researchers to explore and understand the relationship between cardiovascular conditions and PPG signals that were collected using a finger probe.

The final two datasets included in PPG-beats (Charlton et al., 2022a) were collected with the wearable Empatica E4. WESAD (Schmidt et al., 2018) contains data from 15 adults relaxing by a table while reading neutral material, watching a number of amusing video clips, conducting a stress test, and performing mediated recovery. PPG-DaLiA (Reiss et al., 2019) contains data from 15 adults performing the following daily activities according to a protocol: sitting, ascending and descending stairs, playing table soccer, cycling, driving a car, taking a lunch break, walking, and working by a desk.

To analyze PPG signals, data from both healthy and ill people is required. None of the above datasets meets this requirement. In this work, the PPG-BP dataset (Liang et al., 2018a) was selected since it contains the highest number of adult patients, and information on each patient’s disease. Furthermore, it contains data from both young and old adults of both genders. The PPG-BP dataset contains three 2.1 s long PPG segments for each patient. The PPG segments, which were collected with a sampling frequency of 1 kHz, contain 2,100 sampling points. Information on each PPG segment’s Skewness SQI (Ssqi) is reported in the dataset. Only records with a positive Ssqi are included to reduce the artifacts caused by noise and motion (Elgendi, 2016).

### 2.2 System design

**FIGURE 2:**
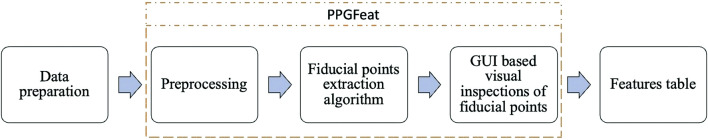
Block diagram of the complete fiducial point detection system.

The developed MATLAB toolbox PPGFeat can automatically identify the fiducial points. The PPGFeat toolbox allows for the application of various preprocessing techniques, such as the use of a filter, smoothing, removing baseline drift, the possibility of calculating PPG derivatives, and implementing algorithms for detecting and highlighting PPG fiducial points. The results can be used to generate more statistically accurate features for further analysis of the PPG signals. The block diagram in Figure 2 outlines the process of analyzing PPG, VPG and APG signals using the PPGFeat toolbox. The process starts with data preparation, where the raw PPG signals are segmented based on their Ssqi. The signals are then subjected to preprocessing, where they are filtered to remove any noise or artifacts. The next stage involves the use of a novel algorithm to extract the fiducial points from the preprocessed signals. The extracted fiducial points are visually inspected to ensure accuracy, and finally, they are used to generate a features table that summarizes the key features of the PPG signals which can be used for further analysis and interpretation.

#### 2.2.1 Data preparation

Data preparation is an essential step in analyzing the online available datasets. It explains the mathematical foundations of the data processing and segmenting the data into raw PPG segments for further analysis.

##### 2.2.1.1 Mathematical foundations

A moving average filter is applied using the MATLAB function *movmean* to reduce random noise and improve the quality of the signal. The governing mathematical equation of the moving average (1) can be written as follows:

$$y\left[i\right]=1/N\sum_{i=0}^{N-1}x_{i+j}$$ (1)

where *x* is the raw PPG signal, *y* denotes the filtered PPG signal, and *N* denotes the average number of points. The derivatives of PPG up to the third level are calculated using the governing Eqs 2–4,

$$VPG=\frac{d}{dt}\left(PPG\right)=\frac{d}{dt}\left[y\left(t+1\right)-y\left(t\right)\right]$$ (2)

$$APG=\frac{d}{dt}\left(VPG\right)=\frac{d}{dt}\left[y\left(t+1\right)+y\left(t-1\right)-2y\left(t\right)\right]$$ (3)

$$JPG=\frac{d}{dt}\left(APG\right)=\frac{d}{dt}\left[y\left(t+2\right)-2y\left(t+1\right)+2y\left(t\right)-y\left(t-1\right)\right]$$ (4)

where *y(t)* is a filtered PPG signal, *y(t+1)* and *y(t-1)* represent the next and the previous sample respectively, and *y(t+2)* represents the 2^nd^ next sample. The VPG, APG and JPG of the denoised PPG signal are calculated using MATLAB’s *diff* function.

##### 2.2.1.2 Data segmentation

**FIGURE 3:**
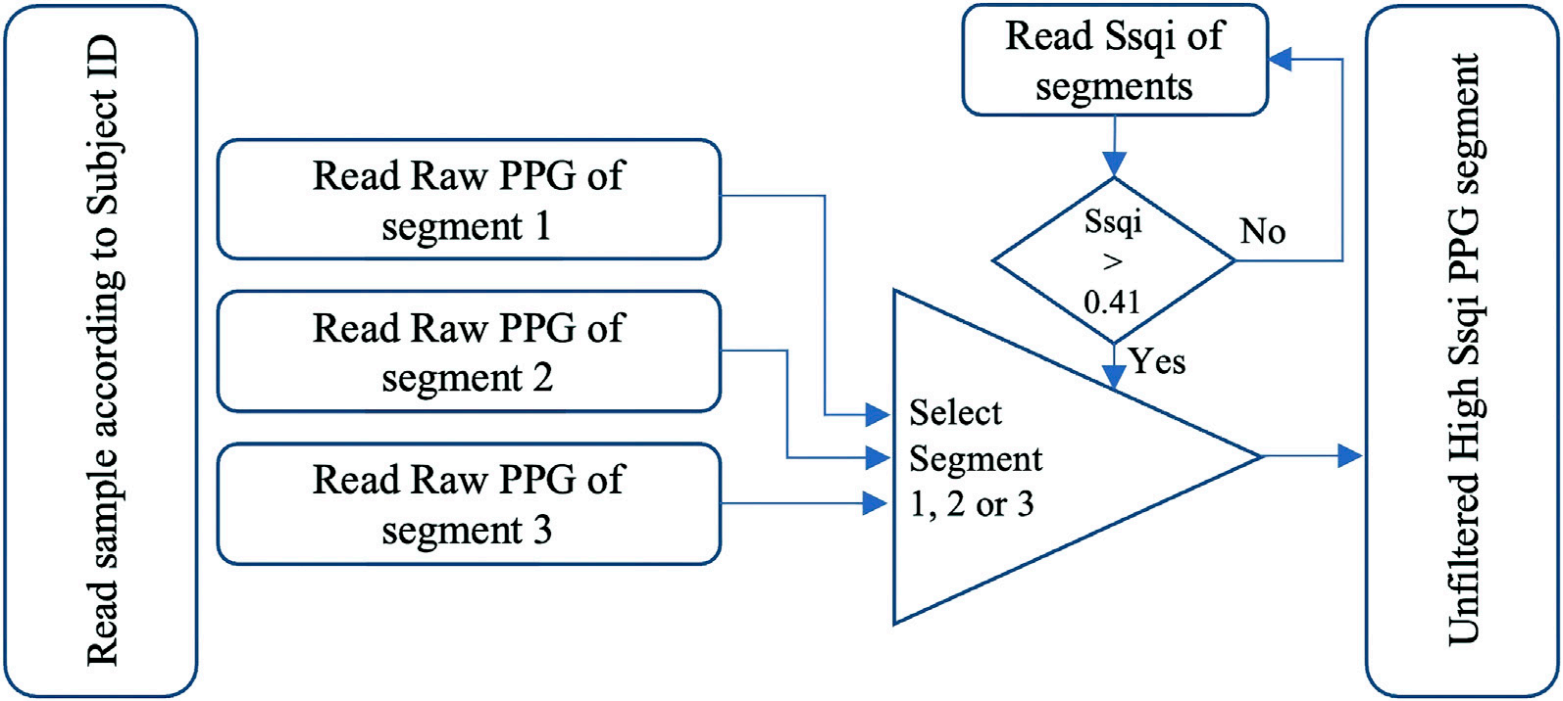
Data selection and preparation process (Abdullah et al., 2023).

Figure 3 illustrates the process of selecting raw PPG segments for each subject in the PPG-BP database (Liang et al., 2018a) using a Ssqi threshold of 0.41. The recorded PPG segments contain 2,100 data points collected during 2.1 s. This process allows for the selection of one single high Ssqi segment for each subject. The raw values of the selected segments are stored in a matrix as shown in the rightmost box of Figure 3, while a second matrix store the corresponding Ssqi values and subject ids. This method is applied to all subjects. It is recommended to generate an input matrix with dimensions *r* x *w*, where *r* represents the number of subjects and *w* represents the PPG data for each subject. Additionally, users of PPGFeat have the option to upload an optional second matrix. This matrix should have dimensions *r* x 2, where column 1 contains the subject ids, and column 2 contains the calculated Ssqi values. The generated PPG segments matrix is then provided as input to the fiducial point extraction process in the PPGFeat toolbox.

### 2.3 PPGFeat toolbox

This section provides an overview of the PPGFeat toolbox GUI and the various steps involved in the PPG data analysis. The PPGFeat toolbox offers a comprehensive set of features, including preprocessing, fiducial point extraction, and visual inspection of fiducial points. In addition, it enables the generation of a PPG features table which can be used for later analysis.

#### 2.3.1 Preprocessing

Previous studies have discussed the importance of choosing the right filter and frequency range for PPG signal analysis (Karlen et al., 2012; Peng et al., 2015; Zhang et al., 2017; Liang et al., 2018c; Huang et al., 2022; Wan et al., 2022). Liang et al. (Liang et al., 2018c) found that a Chebyshev type II 4th order, 20 db filter with a frequency range of 0.4–8 Hz was the most effective in improving the quality of PPG signals. The PPGFeat toolbox uses the same filter design to extract PPG, VPG and APG fiducial points and removes high- and low-frequency noise followed by the moving average filter to further reduce the random noise and improve the signal quality. Furthermore, PPGFeat provides flexibility in the preprocessing settings by allowing users to select different cutoff frequencies for the Chebyshev type II 4th order, 20 dB filter. This is further explained in Section 2.3.3.1.

#### 2.3.2 Fiducial point extraction algorithm

**FIGURE 4:**
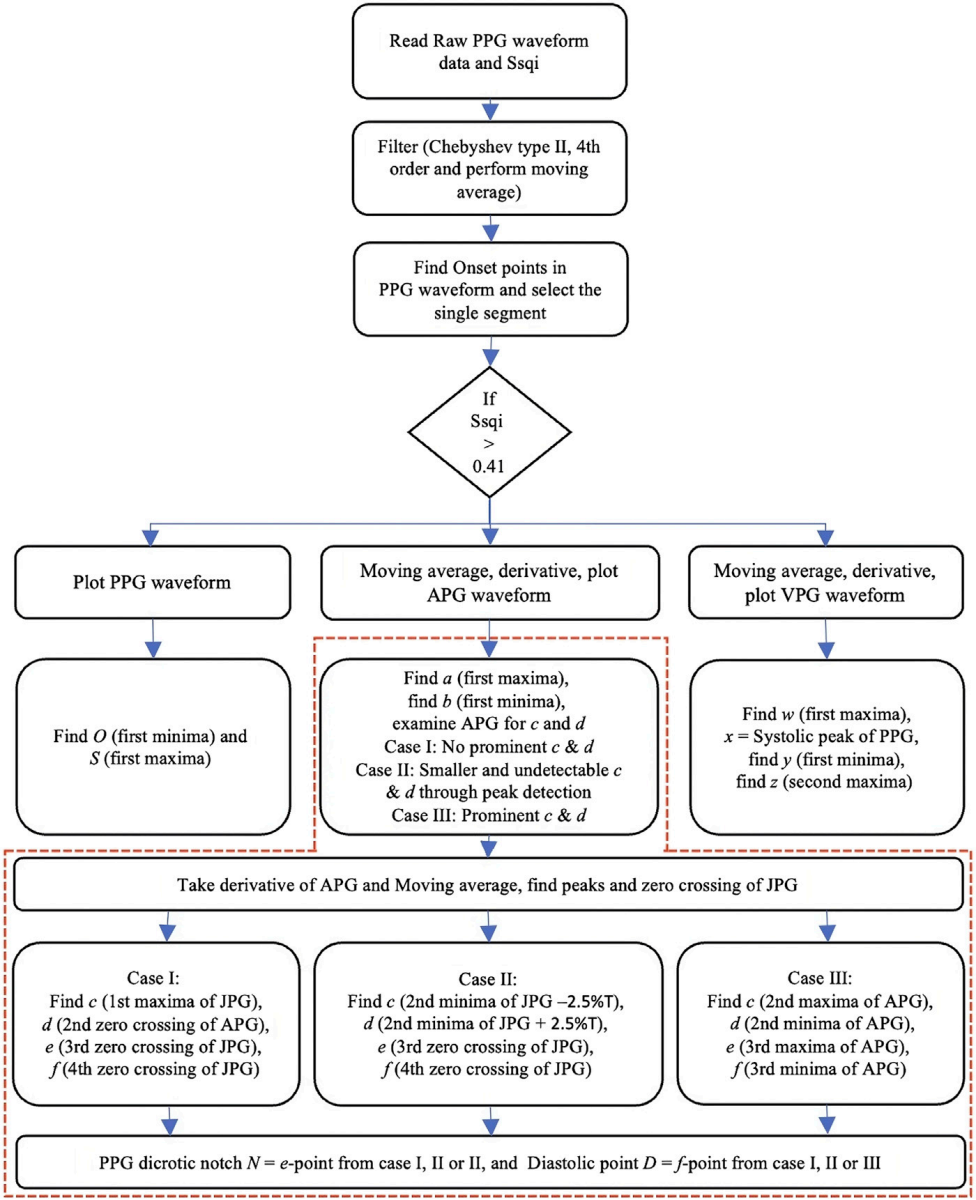
Flowchart of the PPG fiducial point extraction algorithm, the red portion of the flowchart describes the CnD algorithm for APG classification, and the extraction of c and d as described in Abdullah et al. (2023)).

**FIGURE 5:**
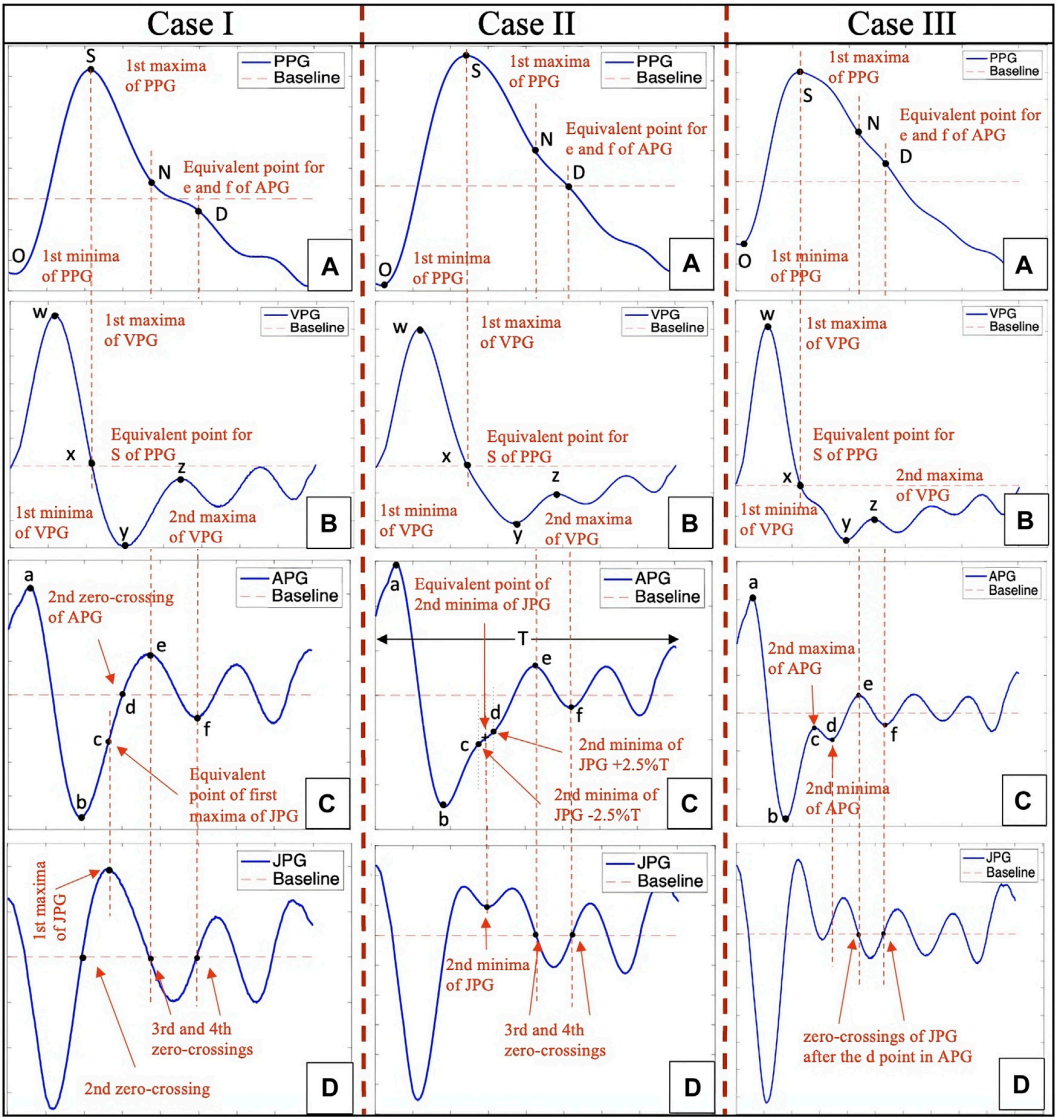
PPG fiducial point extraction through Case I, II and III, where **(A)** is the PPG waveform, **(B)** is the VPG waveform, **(C)** is the APG waveform, which the algorithm examines between the b and e points in order to classify it into Case I, II or III, and **(D)** is the JPG waveform, which is used to extract fiducial points for the APG and PPG waveforms.

The flowchart in Figure 4 presents a comprehensive methodology for identifying fiducial points using filtered PPG, VPG, and APG waveforms. The user first selects the unfiltered high Ssqi raw PPG segments as explained in Section 2.2.1.2 which is then processed through the filtering stage as explained in Section 2.3.1. Second, the selected segment is processed using the MATLAB-functions *islocalmax* and *islocalmin* which identify and highlight the O and S points of the PPG segment, the O points will be used to extract the single PPG segment for further processing. Third, the single PPG segment is differentiated, and a moving average is applied using the MATLAB functions *diff* and *movmean* respectively, to obtain the VPG and APG waveforms. Once all the derivatives of a single PPG segment are obtained, the algorithm starts to locate the fiducial points of the PPG. The O point is the first minima of the PPG whereas the S point is the first global maxima of the PPG. The process is shown in Figure 4 flowchart and Figure 5 (Case I A, Case II A and Case III A). The extraction of the N and D points is explained in Section 2.3.2.1.

The MATLAB functions islocalmax and islocalmin are also applied on the VPG waveform in order to identify four additional fiducial points, i.e., *w* which is the first maxima of VPG, *x* which is the corresponding systolic point of the PPG waveform, *y* which is the first minima of the VPG, and *z* which is the second maxima of the VPG or the zero-crossing point of the APG after the *e* point. The process of extracting the fiducial points is shown in Figure 4 flowchart and Figure 5 (Case I B, Case II B and Case III B).

##### 2.3.2.1 CnD algorithm

The analysis of the APG waveform using the CnD algorithm (Abdullah et al., 2023) begins at the top of the red portion of Figure 4 flowchart. The a point is the first global maxima in the systolic region. This is followed by the first global minima, which is the b point as shown in Figure 5 (Case I C, Case II C and Case III C). However, identifying the c and d peaks in the APG waveforms is challenging due to the variability of their characteristics caused by stationary and non-stationary effects and variations in heart rate. To address this challenge, the algorithm examines the region between the locations of the b and e peaks of the APG waveform and their corresponding location in the third derivative of the PPG waveform, using three distinct algorithms: Case I, Case II, and Case III.

The CnD algorithm (Abdullah et al., 2023) is used to analyze the APG waveform in order to identify the prominent points in the systolic phase (a, b, c, and d) and the diastolic phase (e and f). The algorithm employs different methods depending on how prominent the c and d points are in the APG waveform.

Case I: When there are no prominent c and d points in the APG, as shown in Figure 5 (Case I C), the CnD algorithm examines the JPG waveform (Figure 5: Case I D). This involves locating the zero-crossing points and the first maxima of the JPG waveform that occurs after the second zero-crossing. As shown in Figure 5 (Case I C and D), the first maxima in the JPG waveform corresponds to the c point in the APG waveform, while the APG waveform’s second zero-crossing corresponds to the d point. The e point in the APG waveform corresponds to the third zero-crossing of the JPG waveform or the second maxima of the APG waveform, and the f point corresponds to the fourth zero-crossing of the JPG waveform or the second minima of the APG waveform. The N and D points of the PPG waveform correspond to the e and f points of the APG waveform (Figure 5: Case I A, C and D).

Case II: When the c and d points are undetectable in the APG waveform, as shown in Figure 5 (Case II C), the JPG waveform (Figure 5: Case II D) is analyzed. In this case, the second minima of the JPG waveform is located in the middle of the c and d points. As shown in (Figure 5: Case II C and D), the CnD algorithm calculates the corresponding c and d point in the APG waveform by taking the second minima of the JPG waveform and subtract 2.5% of the total wavelength (T) of the APG waveform for the c point, and add 2.5% T for the d point, respectively. The e point in the APG waveform corresponds to the third zero-crossing of the JPG waveform or the second maxima of the APG waveform, and the f point corresponds to the fourth zero-crossing of the JPG waveform or the second minima of the APG waveform. The N and D points of the PPG waveform correspond to the e and f points of the APG waveform (Figure 5: Case II A, C and D).

Case III: When the c and d points are prominent in the APG waveform, as shown in Figure 5 (Case III C), the CnD algorithm can accurately locate them directly from the analysis of the APG waveform. In this case, the c point is the second maxima of the APG waveform, and the d point is the second minima of the APG waveform (Figure 5: Case III C). The e point in the APG waveform corresponds to the third zero-crossing of the JPG waveform or the second maxima of the APG waveform, and the f point corresponds to the fourth zero-crossing of the JPG waveform or the second minima of the APG waveform. The N and D point of the PPG waveform correspond to the e and f points of the APG waveform (Figure 5: Case III A, C and D).

#### 2.3.3 GUI based visual inspection of fiducial points

**FIGURE 6:**
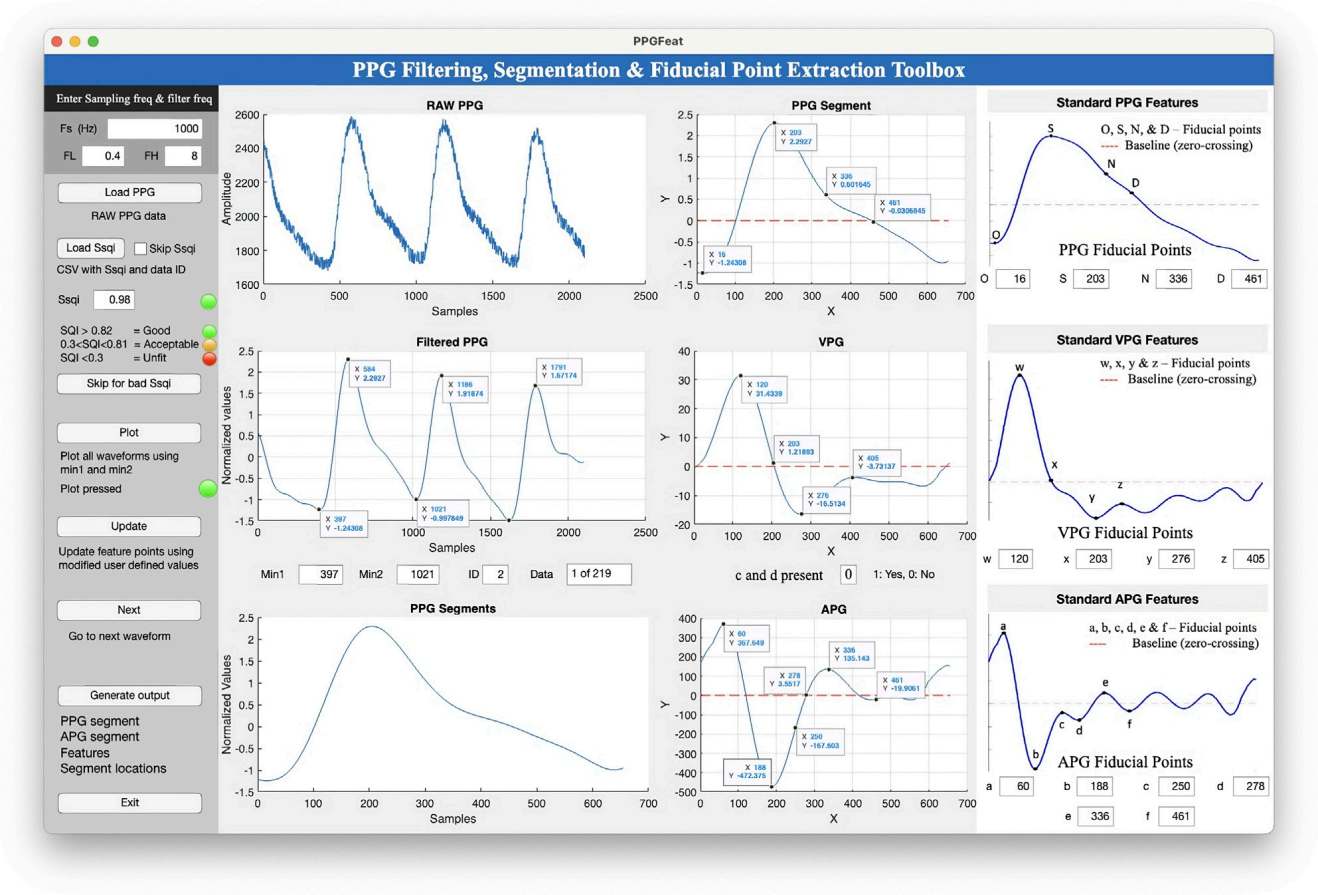
The graphical user interface of the PPGFeat toolbox.

The PPGFeat toolbox GUI (Figure 6) provides an opportunity to select the data preprocessing parameters, visualize the fiducial points and perform manual inspection and adjustments of the extracted fiducial points.

##### 2.3.3.1 GUI

The PPGFeat toolbox GUI shown in Figure 6 is designed to support the user in performing various operations on PPG signals, including filtering, automatically extracting fiducial points, visualizing the fiducial points of the PPG, VPG and APG and generating a features table. The key features of the PPGFeat GUI are.

**FIGURE 7:**
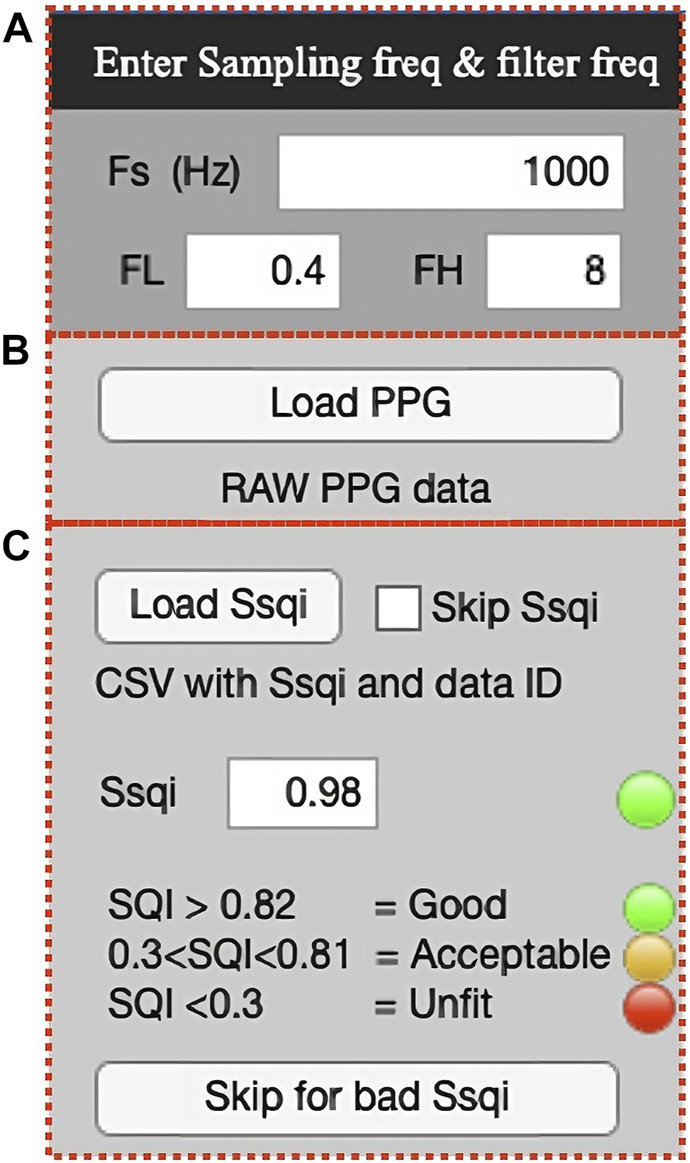
Filter frequency selection **(A)** and PPG and Ssqi data loading **(B, C)**.

**FIGURE 8:**
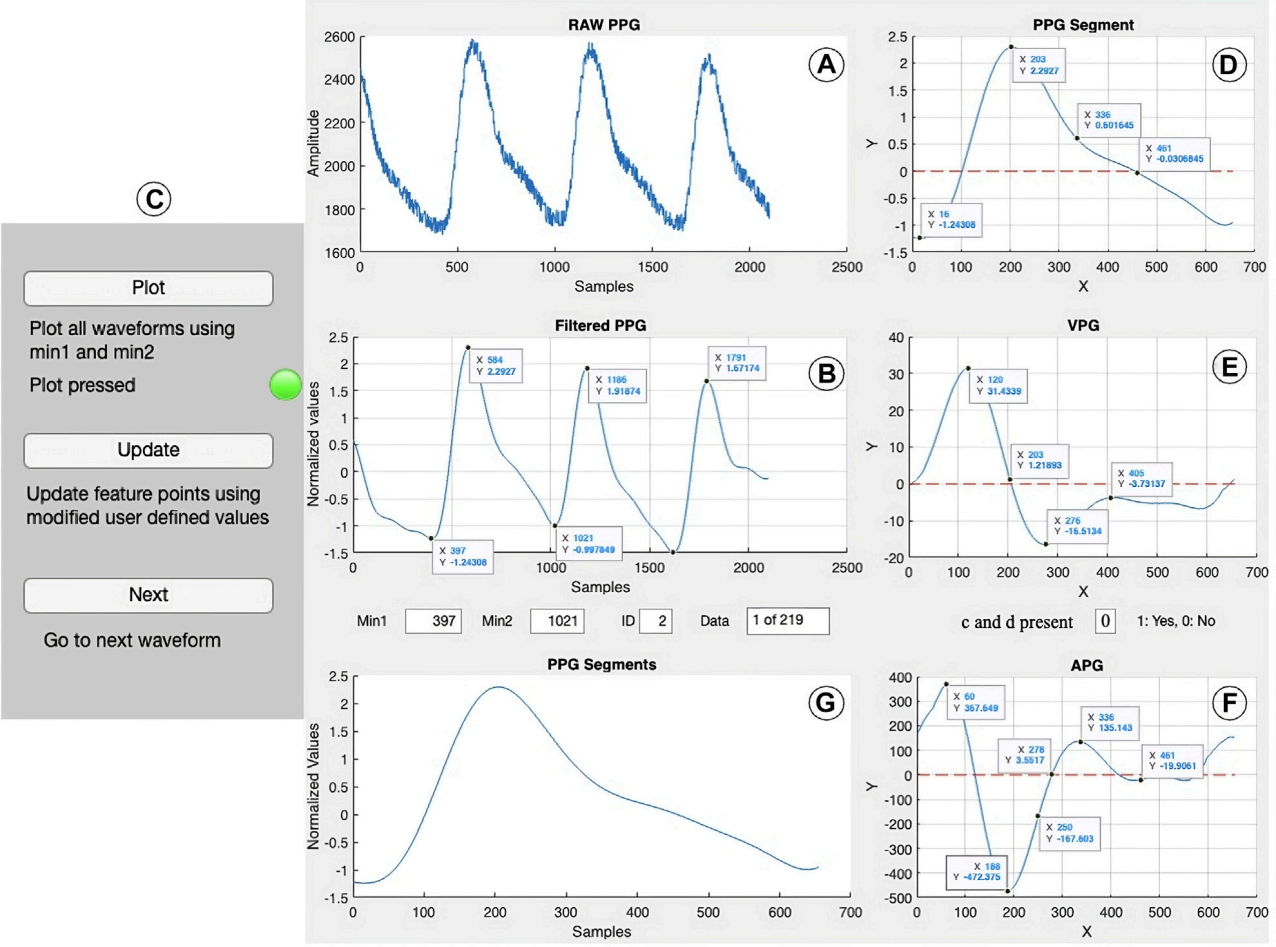
Fiducial point extraction process, where **(A, B)** show the raw and filtered PPG waveform, **(C)** is the GUI control panel, **(D–F)** show the PPG segment and its derivatives along with the extracted fiducial points, and **(G)** shows the single PPG segment of each filtered PPG waveform.

**FIGURE 9:**
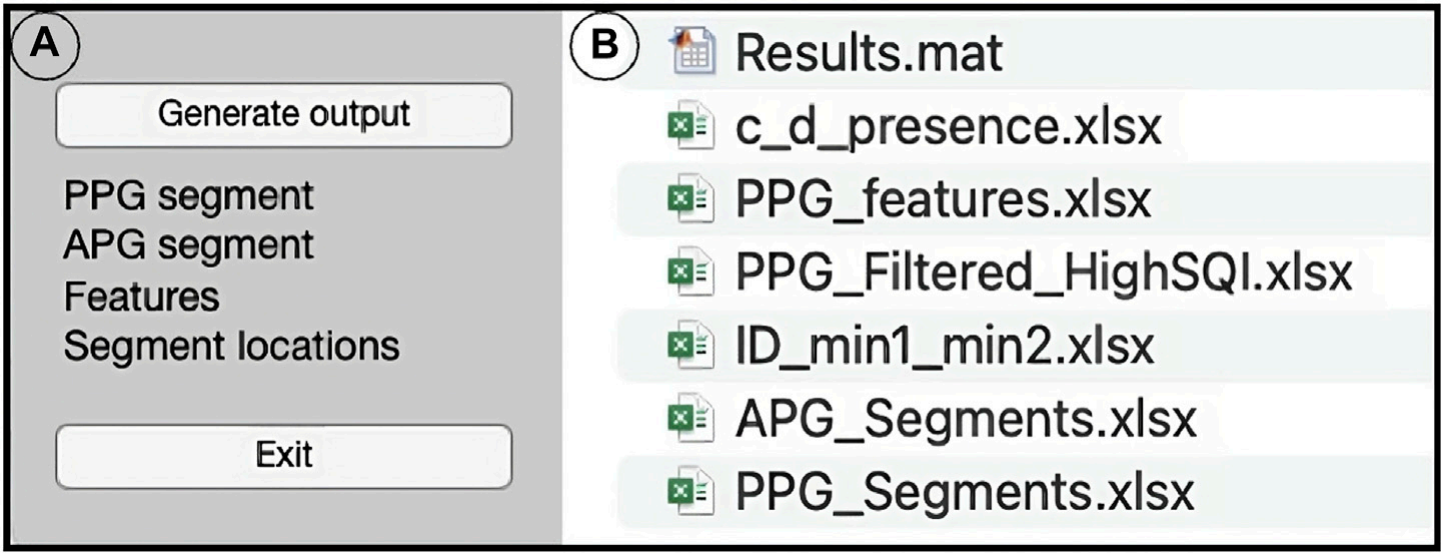
Data storage, where **(A)** includes the “Generate output” and “Exit” buttons and **(B)** shows the generated output files.

1. Filter Frequency: The user is allowed to specify the sampling frequency (Fs) and the bandpass filter frequencies (FL for low-pass and FH for high-pass) for a Chebyshev Type II 4th order filter with a 20 dB attenuation (Figure 7A). This filter is applied to the raw PPG signal in order to obtain the filtered PPG signal.
2. Data Loading: The user can load the raw PPG data of a subject in a comma-separated values (.csv) file by using the “Load PPG” button shown in Figure 7B. In this study, the raw PPG segments were obtained through the data segmentation process explained in Section 2.2.1.2. Additionally, the GUI allows the user to load the Ssqi and data index values of the PPG data using the “Load Ssqi” button, as shown in Figure 7C. If the data index and Ssqi values are not available, the user can select the “Skip Ssqi” option. When developing PPGFeat, the raw PPG data consisted of 219 subjects with 2,100 data points for each subject, resulting in a matrix with the dimensions 219 x 2,100.
3. Fiducial Point Extraction Process: After loading the data, the raw and filtered PPG waveforms are displayed, see Figures 8A, B. Using the filtered PPG, the PPGFeat toolbox locates the starting points of each segment from the PPG (Figure 8B), which are displayed as “Min1″ and “Min2″ in the GUI. The user can change the selected PPG segment by altering the values in “Min1″ and “Min2″, and then plot the single PPG segment and their corresponding VPG, APG segments by clicking the “Plot” button (Figure 8C). The plots, which are shown in Figure 8 D-F, highlight the fiducial points of each waveform. If a fiducial point is incorrectly identified, the user can click the “Update” button in Figure 8C to automatically correct the value and regenerate the plots. To examine the PPG waveform of the next subject, the user can press the “Next” button in Figure 8C. The extracted fiducial points of the current subject will be automatically stored when clicking the “Next” button.
4. Data Storage: After completing the fiducial point extraction process, the user can generate output files by clicking the “Generate output” button in Figure 9A. These files include the filtered and zero-padded data of the PPG and APG segments, PPG segment locations (ID_min1_min2), presence of c and d points, a PPG features table listing the PPG, VPG, and APG fiducial points, filtered PPG waveforms and a MATLAB. mat file containing all generated output files. Table 2 summarizes the details of the output files generated by the PPGFeat toolbox.

**TABLE 2:** Output files from PPGFeat toolbox, where n is the number of samples in the used dataset (n = 219).

File name	Dimension	File type	Content
Results	—	.mat	Combination of all the.xlsx files in the matrix format
c_d_presence	n x 1	.xlsx	0 or 1, where 0 means that c and d were not detected and 1 means detected
PPG_features	n x 30	.xlsx	Time domain and corresponding Magnitude and time domain values of fiducial points for PPG, VPG and APG
PPG_Filtered_HighSQI	n x 2100	.xlsx	Filtered PPG waveforms generated from input data
ID_min1_min2	n x 3	.xlsx	The first column is subject ID, the second and third columns are “Min1″ and “Min2″ values of the selected PPG segment
APG_Segments	n x 1200	.xlsx	Filtered APG segments with zero-padded values
PPG_Segments	n x 1200	.xlsx	Filtered PPG segments with zero-padded values

##### 2.3.3.2 Visual inspection of fiducial points

**FIGURE 10:**
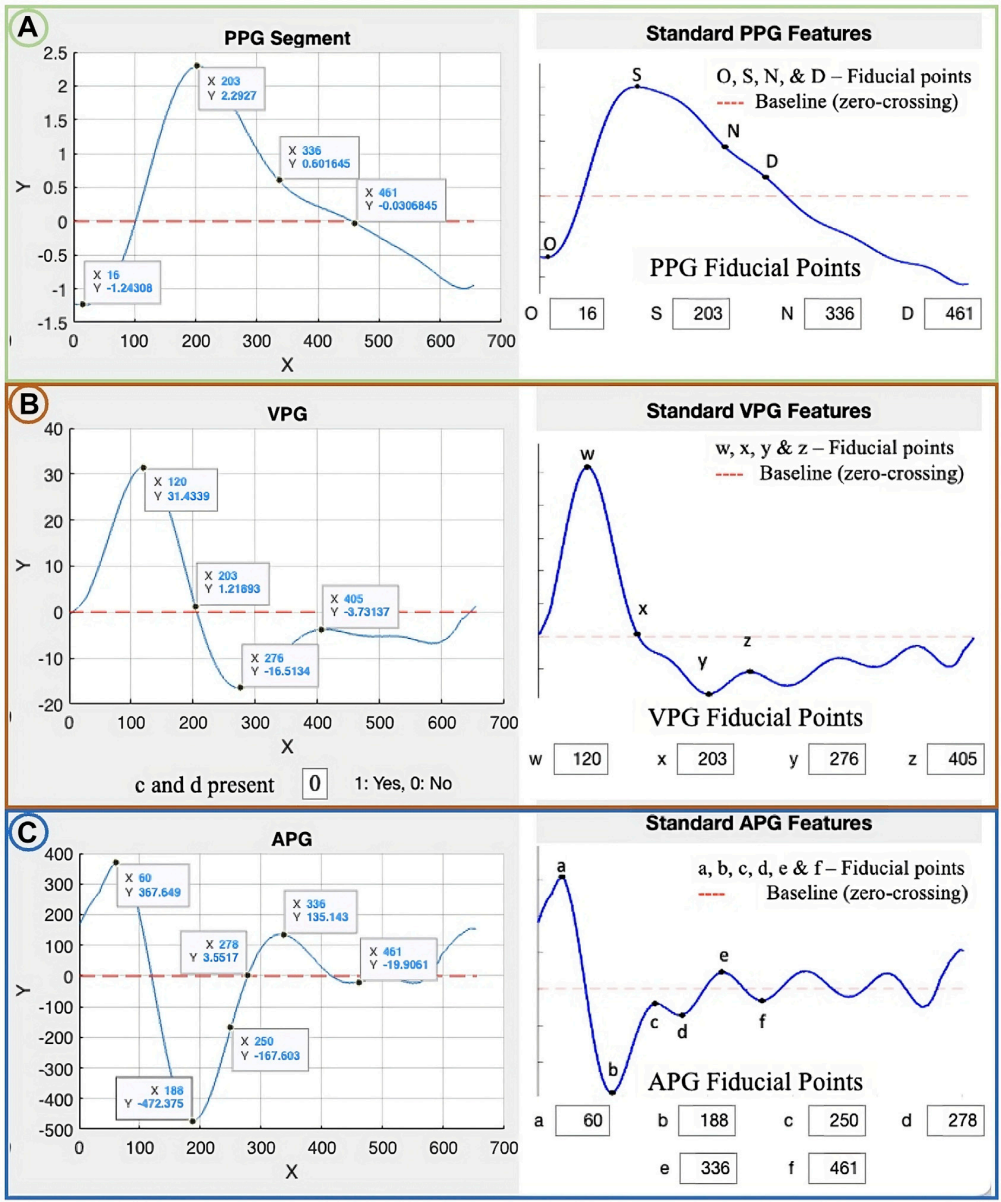
Visual inspection of fiducial points where **(A–C)** show the PPG, VPG and APG waveforms (left) and their fiducial points with respect to standard waveforms (right).

The visual inspection of fiducial points is a crucial step in the analysis of PPG signals. This step allows the user to confirm the accuracy of the extracted fiducial points and to make any necessary adjustments. The PPGFeat toolbox GUI allows the user to examine the PPG, VPG, and APG plots, and to visually inspect the extracted fiducial points as shown in Figures 10A–C. The PPGFeat toolbox highlights the fiducial points on the plots and displays their corresponding time-domain values which makes it easy for the user to identify and adjust the fiducial points if necessary. By conducting a visual inspection, the user can ensure that the extracted fiducial points accurately represent the features of the PPG signals. This will lead to more reliable and statistically accurate results.

### 2.4 Features table

The features table generated by the PPGFeat toolbox provides the fiducial points magnitude and time domain values of the PPG, VPG and APG. A total of 30 features are generated, and include the magnitude features *O, S, N, D, Min2, w, x, y, z, a, b, c, d, e, f,* and time domain features *O_t, S_t, N_t, D_t, Min2_t, w_t, x_t, y_t, z_t, a_t, b_t, c_t, d_t, e_t,* and *f_t*. These features can be used to calculate additional statistical features including, e.g., pulse area (Wang et al., 2009), pulse interval (Poon et al., 2004), augmentation index (Takazawa et al., 1998), and ratios of different fiducial points (Takazawa et al., 1998; Ushiroyama, 2005; Hyun et al., 2007).

The extracted features can be used to determine various physiological parameters such as heart rate (Fu et al., 2008), heart rate variability (Gil et al., 2010), systolic blood pressure (Suzuki and Ryu, 2014), and other cardiovascular parameters (Charlton et al., 2022b). The features can further be used for machine learning, and deep learning to develop predictive models for various cardiovascular diseases (Liang et al., 2018b; Khalid et al., 2018; Subashini et al., 2020; Allen et al., 2021).

## 3 Results

To evaluate the identification and detection of fiducial points, four performance parameters are calculated using Eqs 5–8, which include accuracy (*Acc*), error rate (*Err*), sensitivity (*S*), and positive predictivity (*PP*). Table 3 summarizes the complete results of the performace evaluation.

$$Acc=\left(\frac{TP}{\left(TP+FP+FN\right)}\right)\times 100$$ (5)

$$Err=\left(\frac{\left(FP+FN\right)}{TFP}\right)\times 100$$ (6)

$$S=\left(\frac{TP}{TP+FN}\right)\times 100$$ (7)

$$PP=\left(\frac{TP}{TP+FP}\right)\times 100$$ (8)

**TABLE 3:** Performance evaluation of fiducial points.

Algorithm	N	TFP	TP	FP	FN	S%	PP%	Err%	Acc%
Case I	140	1960	1946	12	2	99.90	99.39	0.71	99.29
Case II	54	756	742	6	8	98.93	99.20	1.85	98.15
Case III	25	350	350	0	0	100	100	0	100

where,

*TP* = True positive (Correctly detected points)

*FP* = False positive (Incorrectly detected points)

*FN* = False negative (Missing points)

*N* = Number of subjects

*TFP* = Total fiducial points

A total of 219 PPG waveforms were processed through the PPGFeat toolbox, and manual marking of fiducial points was performed alongside an automated process using the proposed algorithm. A total of 3066 fiducial points were evaluated out of which 3038 fiducial points were accurately identified by the fiducial point extraction algorithm included in the PPGFeat toolbox, hence a high precision and accuracy in extracting the fiducial points, with an overall accuracy of 99.08% is demonstrated. This is reflected in the accuracy scores for each of the three cases, Case I, Case II, and Case III, which were analyzed separately.

Case I, which accounted for 140 waveforms, showed a fiducial point detection accuracy of 99.29%. Out of 1960 fiducial points in Case I, the algorithm accurately identified 1946, resulting in a sensitivity of 99.90% and a positive predictivity of 99.39%. The overall error rate for Case I was 0.71%. Regarding Case II, which accounted for 54 waveforms, a noticeable presence of c and d points was shown. The algorithm achieved a fiducial point detection accuracy of 98.15%. Out of the total 756 fiducial points in Case II, the algorithm accurately identified 742, resulting in a sensitivity of 98.93% and a positive predictivity of 99.20%. The overall error rate for Case II was 1.85%. Finally, Case III, which consisted of 25 waveforms with a prominent presence of c and d points, showed a perfect accuracy of 100% in detecting the fiducial points. Out of the total 350 fiducial points in Case III, all were accurately identified, resulting in a sensitivity of 100% and a positive predictivity of 100%. The error rate for Case III was 0%.

## 4 Discussion

This article provides information regarding the design and development of the MATLAB based PPGFeat toolbox. The development of the PPGFeat toolbox is motivated by the increasing interest in using PPG signals and their features for various medical applications, including the diagnosis of cardiovascular disease and estimation of systolic blood pressure. However, accurate identification of the fiducial points in the PPG, VPG, and APG waveforms is crucial in order to carry out these tasks with high precision and accuracy. Despite the importance of fiducial point identification, we have not identified any toolbox which is specifically designed for this purpose.

The PPGFeat toolbox was developed using the publicly available dataset PPG-BP dataset (Liang et al., 2018a) which contains PPG waveforms collected from a diverse population including both healthy individuals and individuals with cardiovascular disease or other pathological disorders (Elgendi et al., 2019; Welykholowa et al., 2020). PPG-BP contains a good representation of diverse patients of different ages and with well documented health status, including patients with relevant diseases and healthy individuals. In addition, the Ssqi reported in the PPG-BP dataset makes it possible to reduce the influence of the artifacts in a controlled way.

The PPGFeat toolbox features an interactive, user-friendly and comprehensive solution which facilitates the analysis of the fiducial point extraction using the novel CnD algorithm (Abdullah et al., 2023) on PPG, VPG, and APG waveforms. A major advantage of this toolbox is its ability to evaluate the fiducial points in real-time, which allows the user to validate each waveform individually. Thereby, the risk of errors in the identification of inaccurate fiducial points is reduced. The toolbox also promotes uniformity in existing datasets for evaluating PPG fiducial points, making it a valuable tool for researchers and healthcare professionals.

The PPGFeat toolbox offers an array of signal processing steps, including the selection of the bandpass filter frequencies of a Chebyshev type II 4th order, 20 dB filter, and for different sampling rates. The bandpass filter removes high- and low-frequency noise, while the moving average filter further improves the signal quality by reducing random noise. The toolbox generates a features table comprising time domain and magnitude parameters of the waveforms that can be used for statistical analysis and to determine various physiological parameters such as heart rate (Fu et al., 2008), heart rate variability (Gil et al., 2010), systolic blood pressure (Suzuki and Ryu, 2014), and other cardiovascular parameters (Charlton et al., 2022b). This valuable resource can save researchers time and effort in the preprocessing and analysis of PPG signals.

The performance of the PPGFeat toolbox was evaluated by processing 219 PPG waveforms. The results showed a high performance in extracting the fiducial points, with an overall accuracy of 99.08%. The algorithm’s accuracy was also analyzed for three different cases between which the prominence of the c and d points varies. Case I had a fiducial point detection accuracy of 99.29% and an error rate of 0.71%, Case II had an accuracy of 98.15% and an error rate of 1.85%, and Case III had a perfect accuracy of 100%. These results demonstrate the PPGFeat toolbox’s effectiveness and accuracy in extracting fiducial points from PPG waveforms.

## 5 Conclusion

The PPGFeat toolbox developed in MATLAB is a powerful tool offering an interactive user interface with the ability to accurately evaluate fiducial points in real-time, along with the ability for exporting a comprehensive features table. This makes PPGFeat an attractive option for researchers and healthcare professionals looking to save time and effort in PPG signal analysis. The visual representation of the data provided by the PPGFeat toolbox, along with the ability to store the processed data for conducting further analysis, makes it a unique analysis tool. Furthermore, the features generated by the PPGFeat toolbox have the potential to be used for AI-based predictive analysis, and future work could involve its application on other datasets for further evaluation and comparison.

## Data Availability

Publicly available PPG-BP datasets were analyzed in this study. This data can be found here: https://doi.org/10.6084/m9.figshare.5459299. The PPGFeat toolbox along with documentation, demo video, and preprocessed data are available at https://github.com/saadsur/PPGFeat.
